# Social-Ecological Barriers to Access to Healthcare for Adolescents: A Scoping Review

**DOI:** 10.3390/ijerph18084138

**Published:** 2021-04-14

**Authors:** Whitney Garney, Kelly Wilson, Kobi V. Ajayi, Sonya Panjwani, Skylar M. Love, Sara Flores, Kristen Garcia, Christi Esquivel

**Affiliations:** 1 Department of Health and Kinesiology, Texas A&M University, College Station, TX 77843, USA; kwilson@tamu.edu (K.W.); omo_debare@tamu.edu (K.V.A.); sonya.panjwani@tamu.edu (S.P.); smlove11@tamu.edu (S.M.L.); floressann@tamu.edu (S.F.); kegarcia@tamu.edu (K.G.); c.esquivel@tamu.edu (C.E.); 2 Laboratory for Community Health Evaluation and Systems Science (CHESS), Texas A&M University, College Station, TX 77843, USA; 3 Education, Direction, Empowerment, & Nurturing (EDEN) Foundation, Abuja 900211, Nigeria

**Keywords:** adolescents, healthcare utilization, healthcare access, health services

## Abstract

Access to healthcare for adolescents is often overlooked in the United States due to federal and state-sponsored insurance programs such as Medicaid and the Children’s Health Insurance Program. While these types of programs provide some relief, the issue of healthcare access goes beyond insurance coverage and includes an array of ecological factors that hinder youths from receiving services. The purpose of this scoping review was to identify social-ecological barriers to adolescents’ healthcare access and utilization in the United States. We followed the PRISMA and scoping review methodological framework to conduct a comprehensive literature search in eight electronic databases for peer-reviewed articles published between 2010 and 2020. An inductive content analysis was performed to thematize the categories identified in the data extraction based on the Social-Ecological Model (SEM). Fifty studies were identified. Barriers across the five SEM levels emerged as primary themes within the literature, including intrapersonal-limited knowledge of and poor previous experiences with healthcare services, interpersonal-cultural and linguistic barriers, organizational-structural barriers in healthcare systems, community-social stigma, and policy-inadequate insurance coverage. Healthcare access for adolescents is a systems-level problem requiring a multifaceted approach that considers complex and adaptive behaviors.

## 1. Introduction

Healthcare is essential to the wellbeing of adolescents in the United States (U.S.). Access to healthcare is commonly associated with insurance coverage; therefore, the issue of adolescents’ access to healthcare is frequently minimized due to federal and state-sponsored coverage programs such as Medicaid and the Children’s Health Insurance Program (CHIP). However, healthcare access is more complex than just insurance coverage; it includes the availability of healthcare services, timeliness in treatment, and a competent workforce [1]. The majority of healthcare services are not designed with adolescent patients in mind; therefore, adolescents are at risk of not having a consistent source of care (services), are unable to get care when needed (timeliness), and often lack qualified, competent providers (workforce) that tailor care to their unique needs [2]. Since the implementation of the Affordable Care Act (ACA) in 2010, researchers report that young adults have increased their use of preventive services via well visits (28% pre-ACA to 32% post-ACA) among most racial and ethnic groups [3]. Despite this improvement, less than half of all U.S. adolescents receive well visits, which is key to prevention [3].

Quality healthcare is vital during adolescence because preventive services can modify or deter risky behavior, encourage healthy habits, and promote overall health. Historically, low-income families were more likely to report problems getting their children necessary care and communicating with providers [2]. Youths from low-income families are also less likely to go to primary care providers or have medicine prescribed, but are more likely to use emergency care than middle-to-high-income families [2].

Services tailored specifically to the needs of adolescents are most beneficial. Youth-friendly healthcare services have been successfully embraced in countries with progressive healthcare policies, such as Sweden [4]. In these countries, youth-friendly services are those provided by clinicians who understand and are motivated to work with youths and are located in healthcare settings that ensure confidentiality and embrace a youth-centered approach [4]. These characteristics are important because adolescents often avoid or delay care for sensitive issues due to parental involvement and cite confidentiality as one of the main barriers to their use of healthcare services [4,5]. Furthermore, adolescents are more likely to disclose sensitive health information and return for care in the future if they are assured of confidentiality [5]. Despite this information, studies show that only 30 to 40% of adolescents report spending time alone with their providers during preventive care services, and less than 20% report receiving recommended counseling and screening for high-risk behaviors [6].

Although there is a need to identify barriers that affect youth access to healthcare, there have been no cited review studies that broadly identify these factors within the last ten years. Previous reviews either target specific healthcare services, particular subpopulations and/or include studies outside of the U.S. [7,8,9,10]. Barriers described in these studies focus on mental health services, sexual and reproductive health services, or focus on trafficked youth. While these studies are equally important, a holistic understanding of the barriers that prevent youth access to healthcare in the U.S. is needed.

The purpose of this study was to review the published empirical research on barriers to healthcare access and utilization of services by youths and young adults in the U.S. using the Social-Ecological Model (SEM). The study answered the following research question: “What are the barriers to healthcare access and utilization for adolescents and young adults?”

## 2. Materials and Methods

A scoping review was selected for the study methodology because it enables researchers to identify and map key concepts of existing literature of an under-researched and complex topic [11,12,13] Specifically, Munn and colleagues note that the goal of conducting a scoping review is to “provide an overview or map of the evidence” [11] (p. 3) as opposed to a systematic review, which is used to “produce a critically appraised and synthesized result/answer to a particular question” [11] (p. 3). The scoping review methodology was most appropriate for this study because of the dynamic and complex nature of human development during adolescence, the multifaceted healthcare barriers adolescents encounter, and the abundance of literature investigating adolescent health outcomes across several domains.

This study was guided by Colquhoun and colleagues’ enhanced methodological framework for scoping review [11]. The framework included the following stages: (1) clarifying and linking the purpose and research question(s), (2) identifying appropriate studies, (3) using an iterative team approach for selecting studies and extracting data, (4) incorporating a numerical analysis, (5) summarizing and reporting the study results, and (6) consultation.

### 2.1. Search Strategy

A preliminary search in Google Scholar was conducted in April 2020, which allowed the researchers to test search terms and extract relevant peer-reviewed literature regarding youth’s healthcare access and barriers in the 21st century. With the assistance of a research librarian, a comprehensive search was conducted using eight electronic databases: Child Development & Adolescent Studies, CINAHL Complete, Family & Society Studies Worldwide, Family Studies Abstracts, MEDLINE, ERIC, Mental Measurements Yearbook with Tests in Print, and Psychology and Behavioral Sciences Collection. These databases were selected based on their inclusion of health-focused journals and broader social science content. An updated search was conducted in September 2020 and restricted to the past 10 years (2010 to 2020). Search terms were updated based on the findings from preliminary searches. They included (adolescents or teenagers or young adults or teen or youth) AND healthcare AND (barriers or obstacles or challenges) AND (access to care or access to healthcare or access to services) AND (United States or America or USA or U.S).

### 2.2. Inclusion and Exclusion Criteria

**Figure 1:**
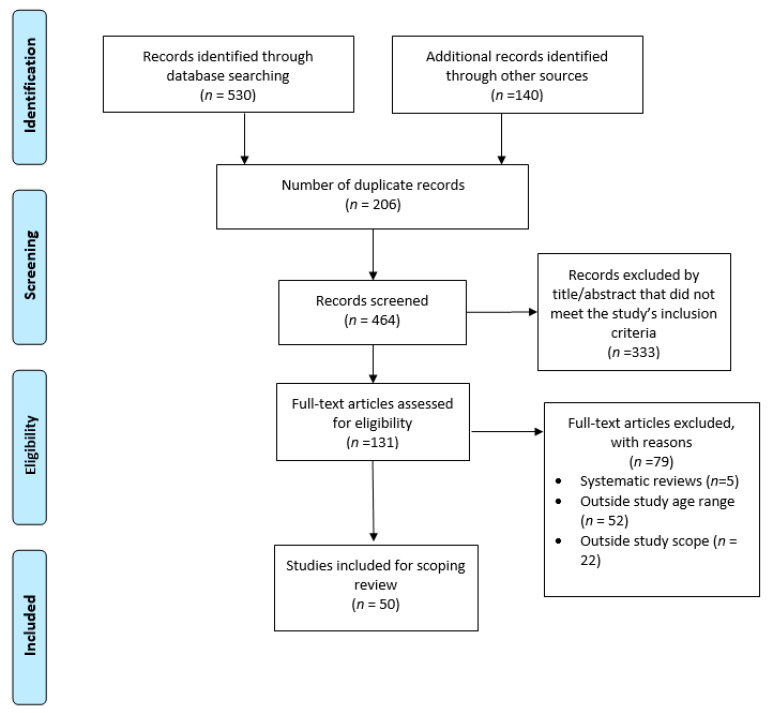
PRISMA flow diagram of included articles for scoping review.

Quantitative, qualitative, and review studies reporting healthcare barriers, access, and utilization among U.S. young adults were included in the review. The included studies represented various healthcare settings, including hospitals, primary healthcare centers/clinics, and school-based health centers. We included studies that analyzed secondary data, evaluated interventions, and reported adolescents’ (10 to 24 years) health outcomes [14]. However, articles with children and young adults were selected if the adolescents’ percentage represented more than half of the study population. We excluded literature reviews and studies reporting outcomes unrelated to healthcare. For this study, we operationalized our study population (adolescents/teenagers and young adults) based on guidelines by the World Health Organization (WHO) and evidence from the literature [14,15]. As such, our study includes articles with persons less than and up to 31 years old, with the majority being during the adolescent years (10 to 24 years). This allowed us to capture all relevant data points about our study population—see Figure 1.

### 2.3. Data Abstraction

Following the scoping review framework, we iteratively abstracted data points using a matrix method [16]. Factors associated with adolescents’ and young adults’ healthcare access and barriers to access were extracted from each study. The screening was conducted in two phases. In phase one, two reviewers independently screened the articles by abstract/title and full text. Extracted points assessed include, but are not limited to, the year of publication, study design, study type, type of care, target population, barriers to access, study setting, and proposed solutions (if mentioned). The raters collaboratively coded the contents using a thematic synthesis and discussed differences identified within the final articles. In phase two, the researchers collaboratively coded the data using a thematic synthesis strategy where key themes identified within the results and discussion of each article were inputted into an Excel database and manually coded to reflect more broader categories. These broad categories were then classified based on their correspondence with the social ecological level of the SEM.

### 2.4. Data Organization Using the Social-Ecological Model

**Figure 2:**
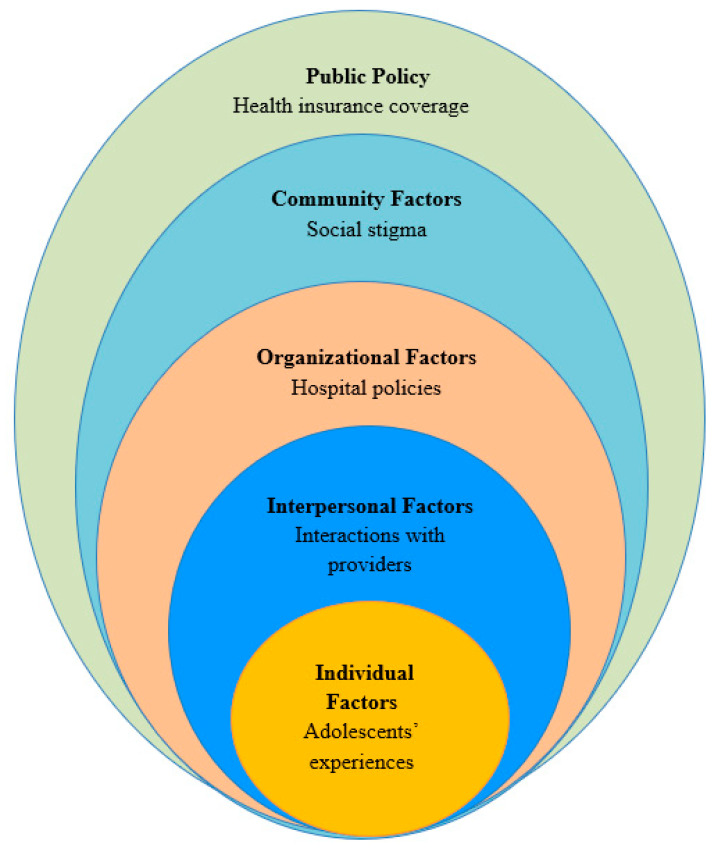
Factors influencing access to care for adolescents.

The SEM posits that individual health behavior influences and is influenced by characteristics within the environment [17]. In this framework, individuals are positioned within multiple hierarchical levels of influence (e.g., intrapersonal, interpersonal, organizational, community, and policy)—see Figure 2. This multifaceted perspective is useful when understanding complex issues such as youth access and healthcare experience. For example, we can use this model to identify and examine relationships between factors that affect youth’s access and experience with services (e.g., knowledge, patient–provider relationships, ability to pay for services, etc.). A social-ecological approach also emphasizes opportunities for comprehensive, multilevel interventions whose effects are more likely to be sustained [18,19].

The Social-Ecological Model for health promotion shows the multilevel factors within the individual, interpersonal, organizational, community, and policy level that affect adolescents’ access to care in the U.S.

## 3. Results

### 3.1. Study Characteristics

Overall, 50 studies were included using an inductive content analysis approach. The majority of studies took place within community-based, primary care settings (i.e., outside of school settings; *n* = 46; 92%). Others took place in school-based health centers and pharmacies (*n* = 2; 4%). Additionally, half of the studies focused on general healthcare (*n* = 25; 50%), a little less than half concentrated on specialized healthcare (e.g., oncology; *n* = 17; 34%), followed by reproductive healthcare (*n* = 6; 12%), and behavioral healthcare (*n* = 2; 4%). The reported health needs in the articles varied. The majority of reported barriers related to youth’s general healthcare needs (*n* = 30; 60%), specialized healthcare needs (e.g., HIV, diabetes, or sickle cell disease; *n* = 19; 38%), and a small number reported the needs of homeless and runaway youths (*n* = 3; 6%). A detailed description of the study’s characteristics is provided in Table 1.

### 3.2. Primary Themes: Barriers to Care

In the following sections, we describe the barriers to youth healthcare access, as seen in Table 2.

#### 3.2.1. Individual Level Factors

Individual level factors, also referred to as intrapersonal factors, are associated with individuals’ characteristics, including knowledge, attitudes, behavior, and skills [17]. In our study, the most cited individual level barriers to access included lack of knowledge of healthcare services and negative beliefs about and experiences with past care (*n* = 18). Some examples include limited knowledge about services, vaccines, and other resources; having prior experience with unmet treatment needs; and personal beliefs about the risk of disease and safety related to vaccines. Navigation of services, including follow-up and adherence, posed the next greatest barrier (*n* = 9). This includes lack of knowledge of steps to take to seek care, attendance at follow-up visits, adherence to prescription medication, and navigation of continuation of care given life transitions. Finally, racial disparities (*n* = 7) and socioeconomic status (*n* = 6) were also reported as barriers to healthcare access for youth.

#### 3.2.2. Interpersonal Level Factors

Interpersonal level factors are characterized by interactions with others, including formal and informal social networks and social support systems; [17] in this study, we examined interpersonal factors between patients and healthcare providers. Cultural and linguistic barriers presented the greatest barrier at the interpersonal level (*n* = 10). These barriers were based on communication challenges, including patients and families with limited English proficiency. Immigration status, cultural differences, and discrimination also contributed to these types of interpersonal barriers. The relationship or lack thereof between patients and providers also posed an obstacle for youths seeking care (*n* = 8). This included poor decision-making on part of the provider, inadequate recommendations for vaccines, and neglect of patients’ physical, mental, and developmental needs.

#### 3.2.3. Organizational Level Factors

Organizational or institutional factors encompass social institutions’ characteristics, including their formal and informal rules and regulations for operation [17]. For this study, we focused on institutional factors within the healthcare system. Of all barriers identified, structural barriers at the organization level were noted as the most significant to impede youth access to healthcare services (*n* = 32). Within this category, limited organizational resources, including drug shortages, clinic space, and shared electronic health records across different types of care, created barriers for adolescent patients when seeking care. Long wait times, lack of organizational regard for barriers to care for youth, and poor coordination of care across different providers also contributed to structural barriers. Financial barriers based on the cost of care were identified as barriers at the organizational level (*n* = 18). A lack of free medical services, families’ inability to afford copays, and high costs of care all contributed to financial barriers. Organizations’ inability to provide confidential services also contributed to barriers youths faced when seeking care (*n* = 8). For example, billing procedures and service statements created privacy concerns for youths because they may reveal health service utilization to parents or caregivers. Lastly, barriers related to physical space within the healthcare setting also prevented youths from engaging in healthcare services (*n* = 4).

#### 3.2.4. Community Level Factors

Within a set boundary, relationships between organizations, institutions, and informal networks comprise community level factors [17]. The primary barrier identified at the community level was the stigma of care-seeking behaviors. Studies report that youths expressed concern with being judged for seeking services (*n* = 8). The stigma around mental health, incarcerated youth, HIV and other sexually transmitted infections, sexual minorities, and sex, in general, were all cited as examples. Transportation to and from clinics, as well as the distance from clinics, also prevented youths from being able to access services (*n* = 7).

#### 3.2.5. Policy Level Factors

Policy level factors include local, state, and national laws and policies [17]. At the policy level, two key themes emerged. The first, insurance coverage (*n* = 20), posed barriers for youths that did not have insurance and even youths with insurance. For youths with insurance, their insurance denied payment or caused delays due to prior authorization requests. This barrier was described with both public and private insurance coverage, limiting young people’s ability to access care regardless of insurance type. Finally, consent policies also impacted youths’ comfort level with seeking care (*n* = 4) primarily because healthcare systems require parent or guardian consent for services. These policies are typically determined at the state level and premised with the idea that parents or guardians seek their child’s best interests. However, more stringent consent laws hinder youths from seeking care due to fear that their parents or guardians will learn about the types of services they desire.

### 3.3. Secondary Theme: Facilitators to Care

Table 3 shows the facilitators to care and proposed solutions based on the social-ecological level. Less than half of the studies assessed proposed solutions to the barriers identified. The most recurring solution required changes to the healthcare system (*n* = 16). Examples include a coordinated care model that improves youth access to different services, using technology to capture health information, establishing culturally and linguistically appropriate service standards, and providing alternative, more easily accessible sites for preventive visits. Outreach was also mentioned as a possible solution (*n* = 8) and included ways to disseminate information related to the types of services offered and the necessity for preventive visits, including vaccines. Studies also discussed youth-friendly strategies to remove barriers to accessing care (*n* = 3). These included web-based technologies that are youth-centered, creating social support systems for youths, and using developmentally appropriate interventions. Lastly, two studies proposed that providing financing options for youths could remove barriers to the cost of care.

## 4. Discussion

This scoping review provides significant insight into the barriers adolescents face when accessing and utilizing healthcare services in the U.S. Given the range of findings across all SEM levels, it is clear that healthcare access for adolescents is a systems-level problem. The complexity of the issue is underscored by multiple influencing factors that are interrelated. Based on the literature, there is no single solution to expanding access for adolescents in the U.S.; rather, a multifaceted approach that considers the complex and adaptive behaviors of interacting factors must be considered. Additionally, our study provided a holistic understanding of the barriers youths face when accessing care, regardless of the context. While we did not differentiate between rural, suburban, and urban settings, the barriers identified in this study can be applied across settings, with the understanding that these barriers may be and are likely augmented in rural settings [70].

The most commonly cited barriers to the utilization of services across the published literature occurred at the organizational level. These barriers were structural and prohibited youths from accessing timely, quality health services. A lack of organizational resources for providing prevention and treatment (drugs, space, technology), long wait times, poor organizational policies, and a lack of coordinated care contributed to these structural barriers. This finding is also supported by the proposed solutions identified, including more coordinated care and applying youth-friendly methods to care provision.

Consistent with the literature, our study also found that the cost of care related to the type or lack of health insurance at the organizational and policy levels presented a significant barrier to obtaining healthcare services [71,72]. It should be noted that the cost of services themselves is not simply what creates these barriers; the capacity to pay for these services also contributes to the issue [73]. The passing of the ACA attempted to address this barrier through the provision of covered preventive health services; however, the structure and financing of adolescent healthcare, including high costs related to treatment, allow inequities in coverage and access to persist, especially among racial and ethnic minorities [74]. Youths need free or low-cost medical services to overcome families’ lack of insurance, inability to afford copays, and overall high costs of care. It should be noted that the cost of care as a barrier to healthcare is not unique to youths alone but a common theme across all age groups in the U.S. For instance, the U.S. ranks higher than most developed nations with seniors (people aged 65 above) unable to access primary care due to cost constraints [72]. Similarly, a large proportion of American adults, irrespective of income levels, reported that the high cost of care makes quality care unaffordable [71]. This indicates that healthcare costs are an inherent barrier to securing quality healthcare in the U.S.

Our study also revealed that several individual-level factors, such as knowledge and awareness of services and cultural beliefs, negatively impacted youths in receiving services and their ability to comprehend treatment and diagnosis. Studies across several health domains and population subgroups have reported similar findings. For example, patients with mental health illness have poor knowledge about available treatment services [6,40]. Moreover, culture plays a significant role in utilizing healthcare services among mentally ill patients [75]. This observation emphasizes the need to address these types of barriers. For example, culturally tailored interventions and innovative health education practices can mitigate disparities to care [76].

Furthermore, our analysis revealed that parental consent laws were a major barrier to youth access and utilization of healthcare services. Consent policies are typically established at the state level and enforced in varying degrees by providers [66]. These laws and the legally defined age for which a minor can provide consent for their care varies significantly by state [77]. For example, concerning abortion services for minors, state mandates and laws differ significantly [78]. Nonetheless, mandates and legislations act as barriers not only to abortion services but also to receiving access to optimal healthcare [79,80].

### 4.1. Future Directions

This scoping review establishes a direction for future research and intervention development. A systems perspective to design interventions that focus on access to healthcare among adolescents is necessary and essential to addressing this public health issue. A systems perspective encourages collaboration among multidisciplinary teams across the social-ecological levels. Multidisciplinary teams that include content experts, providers, and youths themselves must work together to design solutions. Context experts are also important stakeholders that could provide insight into the ways in which local, community-based settings, especially in rural areas, create or remove barriers related to healthcare access. Organizational partners must also be included to address structural barriers to access to improve future interventions. Lastly, interventions must be ecological in their design to address barriers to care adequately. Research shows that designing interventions to improve positive health behaviors among adolescents using the social-ecological framework is effective [81]. Despite these recommendations, caution is also needed. As a systems-level problem, changing any of these influencing factors will impact the entire system. Unintended consequences should be considered in an effort to avoid negative outcomes of interventions.

### 4.2. Limitations

As per the inclusion criteria, studies that included children (<10 years of age) and young adults (>24 years of age) were included in this review. The data from children, adolescents, and/or young adults were pooled, so it was impossible to specifically extract findings targeted at youths or adolescents. This expanded the study’s scope, but the results may not be generalizable for the target population. Researchers attempted to account for this by only including studies where adolescents made up most of the study population. This review also included studies that occurred in highly specific settings and particular subpopulations, which further affected the results’ generalizability. Although this can be limiting, adolescent health needs and issues are heterogeneous, so to adequately capture the most significant barriers faced, it is necessary to pool these findings.

## 5. Conclusions

This study aimed to determine the most cited barriers to youth access to healthcare within the literature. The classification of identified barriers on a social-ecological level brings to light the complexity of this issue and emphasizes that there is not a simple solution to address it. The most cited barriers identified were structural barriers within the healthcare system; barriers posed by policies related to insurance coverage or lack of coverage; individuals’ knowledge, experience, and beliefs about the healthcare system; and financial barriers at the organizational level. The information gleaned from this study indicates that barriers to healthcare access for youths constitutes multiple ecological levels, and thus, requires a holistic approach to promote optimal health for youths and improve their health outcomes later in life. Future research and program models should be systems-based and include multidisciplinary teams to address the complex nature of healthcare access for adolescents.

## Figures and Tables

**Table 1:** Characteristics of Included Articles for Scoping Review.

Author (Year)	Study Design	Healthcare Setting, Type of Care	Study Population, Size	Target Population	Barriers Identified
Okumara et al. (2018) [20]	Quantitative; Cross-sectional	Primary, ^a^ general healthcare	Members of the American Academy of Pediatrics in California, (*n* = 1203)	Children with special healthcare needs	Pediatricians reported a lack of access to mental health services, care coordination, and case management.
Philbin et al. (2014) [21]	Qualitative; Cross-sectional	Primary, other healthcare !	Staff at 15 Adolescent Trails Network clinics, (*n* = 124)	HIV-positive adolescents	Barriers identified included health insurance access, transportation, care coordination, physical space, provider procedural issues, geographic location, and teens’ readiness to care.
Soleimanpour et al. (2010) [22]	Mixed method; Longitudinal	School-based health center, general healthcare	Clients * from 12 school health centers (*n* = 7696), and students (*n* = 105)	Adolescents in middle and high schools	Students do not think they need the needed care, lacked awareness of the school health center’s services, and perceived judgment from peers for seeking care.
Wilkinson et al. (2012) [23]	Quantitative; Cross-sectional	Pharmacy, reproductive healthcare	Pharmacies in 5 cities (*n* = 943)	Adolescents	Adolescent mystery callers received incorrect information about how to obtain emergency contraception.
Goldenberg et al. (2019) [24]	Mixed method; Cross-sectional	Primary, general healthcare	Adolescent Medicine Trials Network for HIV/AIDS Interventions from 14 U.S cities	Black transgender youths (*n* = 110)	Transgender youths with unmet gender affirmation needs had higher shares of anticipated stigma and healthcare nonuse.
Rider et al. (2018) [25]	Quantitative; Cross-sectional	Primary, general healthcare	Minnesota Student Survey (*n* = 80,929; cisgender (*n* = 78,761; TGNC ^+^ (*n* = 2168)	Adolescents in 9th and 11th grades	Gender expression
Macapagal et al. (2016) [26]	Quantitative; Longitudinal	Primary, general healthcare	LGBTQ (*n* = 206)	LGBTQ youths 13 to 24 years old	Insurance access and patient-provider relationships
Baggio et al. (2019) [27]	Quantitative; Longitudinal	Primary, general healthcare	Juvenile offenders (*n* = 4735)	Juvenile offenders 20 to 23	Lack of intermittent health insurance coverage
Luk et al. (2017) [28]	Quantitative; Longitudinal	Primary, general healthcare	Adolescents (*n* = 2023)	Adolescents in 10th grade	Sexual orientation disparity in unmet medical needs was found among males only. On the other hand, sexual minority females were more likely to have no routine checkup in the past year
Chelvakumar et al. (2017) [29]	Quantitative; Cross-sectional	Primary, general healthcare	Homeless and runaway adolescents and young adults from three centers in Central Ohio (*n* = 180)	Homeless and runaway adolescents and young adults	Transportation barriers, health insurance access, cost of care, and issues related to confidentiality and trust with providers
McManus et al. (2013) [30]	Quantitative; Cross-sectional	Primary, general healthcare	Youths with special healthcare needs (*n* = 17,114)	Youths with special healthcare needs (YSHCN) ages 12 and 18.	Gender, race/ethnicity, family income, specific health conditions, not having a medical home, and health insurance are associated with transitioning into adult care.
Tanner et al. (2018) [31]	Mixed method; Longitudinal	Primary, other healthcare	Electronic medical records (*n* = 135), interviews with adolescents (*n* = 28), and adult providers (*n* = 30)	Youths living with HIV	Individual-level barriers (health insurance status and disclosure-related stigma) and structural barriers within the clinic
Valenzuela et al. (2014) [32]	Quantitative; Cross-sectional	Primary, other healthcare	children and youths with type 1 diabetes (*n* = 780)	children and youths with Type 1 diabetes less than 20 years	Cost of care, communication with providers, getting needed information, problems with access to care, not having a regular provider, and receiving contextual care were associated with poorer glycated hemoglobin levels.
Jaacks et al. (2012) [33]	Quantitative; Cross-sectional	Primary, other healthcare	Youths of foreign-born parents with diabetes (*n* = 3086)	Youths of foreign-born parents with Type 1 and 2 diabetes less than 20 years	In the unadjusted analysis, youths with foreign-born parents experienced barriers related to having a regular doctor, access to care, and contextual care
Boulet et al. (2010) [34]	Quantitative; Cross-sectional	Primary, other healthcare	Children with sickle cell disease (*n* = 19,527)	Children with sickle cell disease (SCD) aged 0–17	Black children with SCD experienced problems accessing available healthcare services, such as difficulty setting up an appointment, excessive wait times before seeing a doctor, and difficulty in arranging transportation to the site of a healthcare provider.
Kavanaugh et al. (2013) [35]	Quantitative; Cross-sectional	Primary, reproductive healthcare	Publicly funded family planning facilities (*n* = 584)	Adolescents younger than 20 and young adults aged 20 and 24.	Costs to long-acting reversible contraceptives, inconvenient clinic hours, staff concerns, limited training on implant insertion were barriers to provided contraceptive and long-acting reversible contraceptives.
Ralph & Brindis (2010) [36]	Review	Primary, reproductive healthcare	Not applicable	Adolescents	Common barriers to care for adolescents include concerns about confidentiality and costs.
Strickland et al. (2011) [37]	Quantitative; Cross-sectional	Primary, general healthcare	Adolescents (*n* = 83,448)	Adolescents aged 1–17	Disparities to having a medical home included race/ethnic characteristics, socioeconomic status, and existing health conditions.
Islam et al. (2019) [38]	Quantitative; Cross-sectional	Pharmacy, reproductive healthcare	Licensed pharmacists in eight states (*n* = 40)	Adolescents 9–17	Challenges to HPV vaccination included parental consent, tracking and patient recall, perceived stigma of immunization, and promotion of vaccination
Minnaert et al. (2020) [39]	Quantitative; Cross-sectional	Primary, general healthcare	Children with hearing difficulties (*n* = 40,242)	Children with hearing difficulties aged 0–17	Children with hearing difficulties did not have access to a medical home, community services, and adequate health insurance coverage
Dang et al. (2011) [40]	Qualitative; Cross-sectional	Primary, general healthcare	Youths and young adults enrolled in a Healthshack-a personal health information system (*n* = 149)	Runaway and homeless youths and young adults ages 9 to 24.	Access and knowledge about care, inconsistence use of medications, lack of medical follow-ups, and transportation issues were reported barriers youths encountered
Lai et al. (2016) [41]	Qualitative; Cross-sectional	School-based health center, behavioral healthcare	Mental/primary care providers and care coordinators from 14 SBHCs (*n* = 43)	Not specified	Providers reported that concerns about trust, confidentiality, and stigma related to mental illness inhibit the use of services among students.
Hallum-Montes et al. (2016) [42]	Qualitative; Cross-sectional	Health centers, reproductive healthcare	Staff members of 30 health centers in 7 States (*n* = 85)	Adolescents	Structural barriers within the health system and community-related factors prevented implementation of evidence-based clinical practice for adolescent’s reproductive healthcare.
Mullins et al. (2016) [43]	Qualitative; Cross-sectional	Primary, other healthcare	Clinicians from the Adolescent Medicine Trials Network for HIV/AIDS Interventions (ATN) in 14 U.S. locations (*n* = 15)	HIV-infected and at-risk adolescents and youths	Barriers to prescribing PrEP to minors and youths were categorized in the patient-level, provider-level, organizational/systems-level, and community-level factors.
Connors (2019) [44]	Review/Case study	Primary, general healthcare	Not applicable	Adolescents and youths	Latino children and their families face barriers related to limited English proficiency, poor understanding of the U.S. healthcare system, lack of providers, and immigration status complications.
Ozturk et al. (2014) [45]	Quantitative; Cross-sectional	Primary, other healthcare	Teenagers and young adults	Teenagers and young adults aged 15–24 with muscular dystrophy	Race is correlated with healthcare utilization. Blacks have lower overall utilization, less primary/therapy/specialist care, and higher emergency department utilization than other races
Miller et al. (2019) [46]	Quantitative; Longitudinal	Primary, other healthcare	Data from eight adolescent medicine clinical trial units (*n* = 2,142). Key informants (youths, *n* = 39; adults, *n* = 152).	Youths aged 12–24 newly diagnosed with HIV	Barriers addressed included linkage to care, the continuation of care, structural barriers, youth-friendly services, and stigma.
Grossbard et al. (2013) [47]	Quantitative; Cross-sectional	Primary, general healthcare	Young adults (*n* = 27,471)	Young adults (veterans and civilians) aged 19–30	Gender differences in healthcare access and utilization. Women were more likely to have health insurance and being able to see a provider than men
Hudson et al. (2010) [48]	Qualitative; Cross-sectional	Primary, general healthcare	Homeless youths (*n* = 24)	Homeless youths aged 18–25	Homeless youths experience structural barriers within the health system and social barriers, including law enforcement and society.
Marks et al. (2017) [49]	Quantitative; Cross-sectional	Primary, other healthcare	Young men who have sex with men (*n* = 2297)	Young HIV uninfected men who have sex with men aged 18 –24	Lack of access to care differed by and associated with age, race/ethnicity, education, and region.
Bessett et al. (2015) [50]	Qualitative; Cross-sectional	Primary, reproductive healthcare	Young adults (*n* = 89)	Young adults aged 18–26	Young adults seeking contraceptive care had low health insurance literacy and faced barriers related to information and privacy
Anderson et al. (2018) [51]	Quantitative; Cross-sectional	Primary, general healthcare	Pediatric patients (*n* = 98)	Pediatric patients with appendicitis	Structural barriers relating to practitioner misdiagnosis, lack of health insurance coverage, no frequent pediatrician, cost of care, limited knowledge of appendicitis
Smalley et al. (2014) [52]	Quantitative; Cross-sectional	Primary, general healthcare	Children with special healthcare needs (*n* = 40,242)	Children with a special healthcare need aged 0–17	Families of children with greater functional limitations were less likely to make a shared-decision with their providers. Low socioeconomic status and race were associated with low shared-decision making attainment rates
Kreider et al. (2016) [53]	Quantitative; Cross-sectional	Primary, general healthcare	Children with household income between 100% and 300% of the federal poverty line (*n* = 80,655)	Children from low-income households aged 17 and younger	Access to specialty care, inability to obtain healthcare services, those with special healthcare needs, and health insurance type.
Mason et al. (2013) [54]	Quantitative; Cross-sectional	Primary, behavioral healthcare	Young adults aged (*n* = 14,718)	Young adults aged 18 to 23	Gender, substance use, and race/ethnicity were associated with perceived mental health treatment needs.
MacQueen et al. (2015) [55]	Qualitative; Cross-sectional	Primary, other healthcare	Young adults (*n* = 508)	Young Black adults aged 18–30	Barriers to receiving HIV testing were related to perceived risk and stigma. Low-income Black adults experience reduced access to healthcare services.
Avila & Bramlett (2013) [56]	Quantitative; Cross-sectional	Primary, general healthcare	Adolescents (*n* = 91,642)	Adolescents aged 0–17	Immigration status and non-English speaking household as primary language was associated with disparities to care, dental health, consistent insurance, and having a medical home among first-generation vs. non-immigrant Hispanic children, non-immigrant Hispanic children vs. non-Hispanic white children, and Hispanic children in English speaking household vs. non-Hispanic white children.
Kruszka et al. (2012) [57]	Qualitative; Cross-sectional	Primary, general healthcare	Former foster youths (*n* = 9)	Uninsured former foster youths	Former foster youths reported issues relating to not having the right documentation, roadblocks to securing healthcare insurance, and lack of knowledge about Medicaid eligibility.
Kubicek et al. (2019) [58]	Qualitative; Cross-sectional	Primary, other healthcare	Young Black men who have sex with men (*n* = 49)	Young Black men who have sex with men aged 16 to 24	Limited health literacy, inability to identify appropriate providers, cultural values, and histories concerning healthcare and cultural competency among community providers were reported barriers to care among Black young men who have sex with men
Sudhinaraset et al. (2017) [59]	Qualitative; Cross-sectional	Primary, general healthcare	Undocumented Asians and Pacific Islanders (*n* = 32)	Undocumented Asians and Pacific Islanders aged 18–31	Financial costs associated with healthcare services were major barriers undocumented immigrants experienced pre-DACA period. DACA ineligibility for family members prevented others from seeking the needed healthcare services
Monz et al. (2019) [60]	Quantitative; Cross-sectional	Primary, other healthcare	Caregivers of children with autism (*n* = 10,123)	Children with autism aged 3–17 years	Caregivers reported that waiting-list, no-coverage, and costs were common provider and health plan-related barriers. Waiting-list was common in metropolitan areas than non-metropolitan areas.
Kelly et al. (2019) [61]	Quantitative; Cross-sectional	Primary, other healthcare	Pediatric oncologists (*n* = 18)	Pediatrics, adolescents, and young adults	Pediatrics oncologists reported delay in prior authorization requests created a delay in receiving planned chemotherapy, and supportive care treatment and medication access were associated with a delay in starting therapy.
Lin et al. (2013) [62]	Quantitative; Cross-sectional	Primary, other healthcare	Youths with Type 1 Diabetes (*n* = 1012)	Youths below 19 years with Type 1 Diabetes	Racial/ethnic group, insurance status, and household income appeared to influence whether participants were switched from injection to pump therapy
Berg et al. (2016) [63]	Qualitative; Cross-sectional	Primary, other healthcare	Healthcare providers of young adult cancer survivors (*n* = 21)	Adolescents, young adult cancer survivors	Systems-level barriers to engagement in survivorship care included limited resources, role confusion, communication challenges, and lack of insurance coverage. Patient-level barriers include psychological barriers, resistance to survivorship care, and physical barriers.
Calderon et al. (2017) [64]	Review	Primary, general healthcare	Parental consent laws for oral health	Adolescents	Barriers to quality care for adolescents are related to variation and lack of clarity in state laws, a strict opt-in approach to obtaining parental consent, and lack of evidence-based approach to determine adolescents’ cognitive ability to consent.
Cheak-Zamora et al. (2013) [65]	Quantitative; Cross-sectional	Primary, other healthcare	Youths with and without autism spectrum disorder (*n* = 19,004)	Youths with and without autism spectrum disorder aged 12–17	Among youths with an autism spectrum disorder, race/ethnicity and multiple health conditions were associated with not receiving healthcare transitioning services.
Bernstein et al. (2016) [66]	Qualitative; Cross-sectional	Primary, other healthcare	Administrators from 6 clinics in 2 states (*n* = 39)	Pediatrics	Administrators reported that limited time, lack of training and expertise, low caregiver literacy, and lack of shared medical and dental electronic records inhibited cooperation for quality oral healthcare
Kaplan (2010) [67]	Review	Primary, general healthcare	Not applicable	Adolescents	Low utilization of preventive and acute services, inadequate or no health insurance, behavioral issues, financial barriers, and parental perspectives about vaccines are associated with vaccination uptake.
Deutsch & Fortin (2015) [68]	Review	Primary, general healthcare	Not applicable	Children in foster care	Children in foster care experience barriers to receiving quality care related to factors precipitating their removal from care, including chronic neglect of their physical health, mental health, and developmental needs.
Keeton & Chen (2010) [69]	Review	Primary, general healthcare	Not applicable	Adolescents	Barriers to immunizations include infrequent preventive visits, incomplete records, lack of awareness about the risk of serious infectious diseases, and lack of coverage for adolescent vaccination

* provider-reported clinic clients. ^+^ Transgender and gender nonconforming youth. ! Includes other domains of care (HIV or dental). ^a^ The primary care setting includes healthcare provided by any medical professional, including community-based or care networks.

**Table 2:** Barriers to Access to Care for Adolescents according to the Social-Ecological Model.

Ecological Level	Themes	Subtheme	Number of Articles
Individual	Diversity		10
		LGBTQ	3
		Race	7
	Navigation		9
	Socioeconomic status		6
	Behavioral health		5
	Experiences/knowledge/belief		18
Interpersonal	Lack of youth-friendly services	Patient–provider relationship	8
	Cultural/linguistic barriers		10
Organizational	Healthcare system/structural barriers		32
	Financial	Cost of care	18
	Lack of youth-friendly services		12
		Confidentiality/trust	8
		Physical space	4
Community	Stigma		8
	Transportation		7
Policy	Lack of youth-friendly services	Parent consent policy	4
	Financial	Health insurance	20

**Table 3:** Facilitators to Access to Care for Adolescents.

Ecological Level	Themes	Number of Articles
Organizational	Changes to the Healthcare system	16
Organizational	Outreach	8
Organizational	Youth-friendly services	3
Organizational	Cost	2

## Data Availability

Not applicable.
